# People with Parkinson’s perspectives and experiences of self-management: Qualitative findings from a UK study

**DOI:** 10.1371/journal.pone.0273428

**Published:** 2022-09-09

**Authors:** Ria Shah, Joy Read, Nathan Davies, Danielle Nimmons, Jennifer Pigott, Anette Schrag, Kate Walters, Megan Armstrong

**Affiliations:** 1 Department of Primary Care and Population Health, University College London, London, United Kingdom; 2 Institute of Neurology, University College London, London, United Kingdom; Flinders University, AUSTRALIA

## Abstract

**Introduction:**

Parkinson’s prevalence is growing, and more people are being impacted by the condition than ever before. Self-management has been proposed as one way to enable people living with the condition to improve or maintain their quality of life and wellbeing whilst living at home.

**Aim:**

To explore the views and experiences of how people living with Parkinson’s self-manage their condition and identify areas needed to be incorporated into self-management resources or interventions.

**Method:**

Twenty people with Parkinson’s from across London and Hertfordshire, UK took part in semi-structured interviews on self-management. Interviews were transcribed and analysed using thematic analysis to identify themes.

**Results:**

Three main themes were identified: (1) Management of physical symptoms, which included engaging in physical activities, adapting their lifestyles, managing medication and using e-health resources; (2) Management of emotional impact, which involved using a range of cognitive and practical strategies, and seeking talking therapies and medication; and (3) barriers to self-management such as accessing accurate information, experiencing stigma towards their condition which impacted their self-esteem and identity, in turn impacting on their ability to self-manage.

**Conclusion:**

Holistic and person-centred self-management programmes or interventions should be developed incorporating components such as medication and emotional support, individualised planning of exercise regimes, and accessible, timely and accurate information. Furthermore, more public health knowledge on Parkinson’s is needed to help reduce stigma.

## Introduction

Parkinson’s disease prevalence has increased worldwide from 2.5 million in 1990 to 6.1 million in 2016 [1] with the amount of people with Parkinson’s set to rise to 12 million by about 2050 [2]. Symptoms of Parkinson’s include a range of motor problems such as gait, balance issues, slowness, stiffness, and tremor; non-motor symptoms can also occur which consist of disturbance of mood, and cognitive, autonomic, sensory and sleep disorders [3]. With progressing disease, complications such as fluctuations, dyskinesia, and on-off episodes can occur [4]. Parkinson’s symptoms compromise health-related quality of life by making it difficult for people with Parkinson’s to participate in valued activities and roles of home and community life [5]. One way to support people with Parkinson’s is through supporting them in the self-management of their symptoms.

Self-management is defined as a dynamic, interactive, and daily process which individuals engage in to manage chronic illnesses [6]. Such management may include taking medications, making lifestyle changes, or undertaking preventative action [7]. While self-management involves individuals having knowledge, skills, and confidence in managing daily tasks and living well with their chronic condition, healthcare professionals also play a role in supporting self-management skills in their patients. They provide education and supportive interventions to increase patient’s skills and confidence in managing their health problems [8]. Successful self-management has been said to be influenced by self-efficacy [9]; therefore, understanding the needs of those affected by illness, and their capability to self-manage is important, to efficiently support them.

Most research on self-management has focused on chronic conditions such as arthritis and diabetes [10]; however, there is a growing focus on Parkinson’s [11, 12]. Parkinson’s self-management includes adherence to medication regimes, exercising, diet management, the monitoring of various symptoms, and management of environmental factors [13]. A systematic review exploring the effectiveness of self-management interventions found mixed results due to small and poor-quality studies [11]. However, components more frequently observed in effective interventions were information about resources; training or rehearsing psychological strategies; social support; and lifestyle advice and support. This finding is in line with a systematic review of qualitative research with people with Parkinson’s and their carers on self-management, which found these components were viewed as important by people living with Parkinson’s [12]. However, in addition, people with Parkinson’s highlighted needing self-monitoring techniques (e.g., training or aids to do so) and support managing their medication. Importantly alongside all these various self-management components they wanted to maintain their independence and have an individual approach tailored to different stages of the disease. The findings found in people with Parkinson’s and their carers were echoed in a study interviewing health care professionals about self-management [14].

While the systematic review of qualitative evidence [12] provides some evidence with which to develop self-management techniques or interventions, the primary studies have been in a limited range of settings, namely Sweden, Portugal, New Zealand, Canada, and USA and may not be applicable elsewhere, such as in the UK. People’s ability to self-manage is context based due to differing health and social care systems and attitudes towards self-management. Self-management studies have been criticized for placing too much emphasis on the individual with the condition and not exploring wider context and relationships between patients and health care professionals [15]. Therefore, the aim of this paper is to explore how people with Parkinson’s within the UK self-manage and the barriers to doing so, including both individual and wider contextual factors.

## Method

This study was conducted as part of a larger programme of work developing a co-designed, facilitated self-management toolkit for people with Parkinson’s.

### Design

A qualitative design with individual semi-structured interviews was used. Reporting is guided by the Standards for Reporting Qualitative Research framework [16].

### Sampling and participants

Participants were recruited during January to July of 2019, using hospital outpatient clinics and General Practice surgeries in Greater London and Hertfordshire. Purposive sampling based on age, gender, and disease stage was used to recruit participants. Initial approaches for participant recruitment were made by clinicians of NHS Parkinson’s services and primary care; potential participants were given invitation letters and information sheets which they were asked to return to the research team should they wish to take part in the study. Following this, eligibility of participants was confirmed and written informed consent was obtained. Exclusion criteria included: people with Atypical Parkinsonism, those who lived in care homes, anyone lacking capacity to provide informed consent to participant, and those with a life expectancy of less than six months.

### Ethics

This study was given a favourable opinion by the London Queen Square Research Ethics Committee (18/LO/1470).

### Procedure

Interviews were semi-structured and followed a topic guide that covered topic areas such as barriers to self-managing, techniques to manage symptoms, and suggestions for a self-management resource. Prompts and probes were used to facilitate in-depth responses. The interviews were carried out by a team member (JR), who has a background in nursing and psychology, and a research assistant who has a background in psychology. Interviews lasted up to 90 minutes and were audio-recorded.

### Analysis

A constructionist thematic analysis, guided by Braun and Clark’s framework [17, 18], was used to identify, analyse, and report themes. An inductive approach was used to carry out the thematic analysis and to create themes that provide an insight into participants’ perspectives of their experiences. Line-by-line coding was undertaken using NVIVO 20 [19]. An initial coding framework was developed independently by the first author (RS), a Population Health MSc student, consisting of potential themes that were discussed with two authors (MA and JR) and refined. MA has a background in Psychology and is a Senior Research Fellow and JR is a programme coordinator with a background in Nursing and Psychology. The framework was then applied to the transcripts. Themes and their meaning and interpretations were developed further iteratively in a series of discussions with the team (all authors). The study authors are multidisciplinary and consist of researchers with backgrounds in psychology and ageing, and health care professionals including general practitioners and neurologists.

## Results

Twenty participants were recruited including a mix of genders, ages and length of living with Parkinson’s. See Table 1 below for more detail.

**Table 1:** Characteristics of participants (n = 20).

Participants		
**Gender**	Men	12
	Women	8
**Age**	Mean–years	72 years
	Range–years	45–89 years
**Ethnicity**	White British	16
	Asian/Asian British	4
**Paid Carers**	Yes	3
	No	17
**Duration since Parkinson’s diagnosis**	Mean—years duration	8 years
	Range–years duration	5 mo– 20 yrs
**Dementia**	Yes	3
	No	17
**Indices of deprivation**	Mean	6.8
	Range	3–10

### Themes

**Fig 1:**
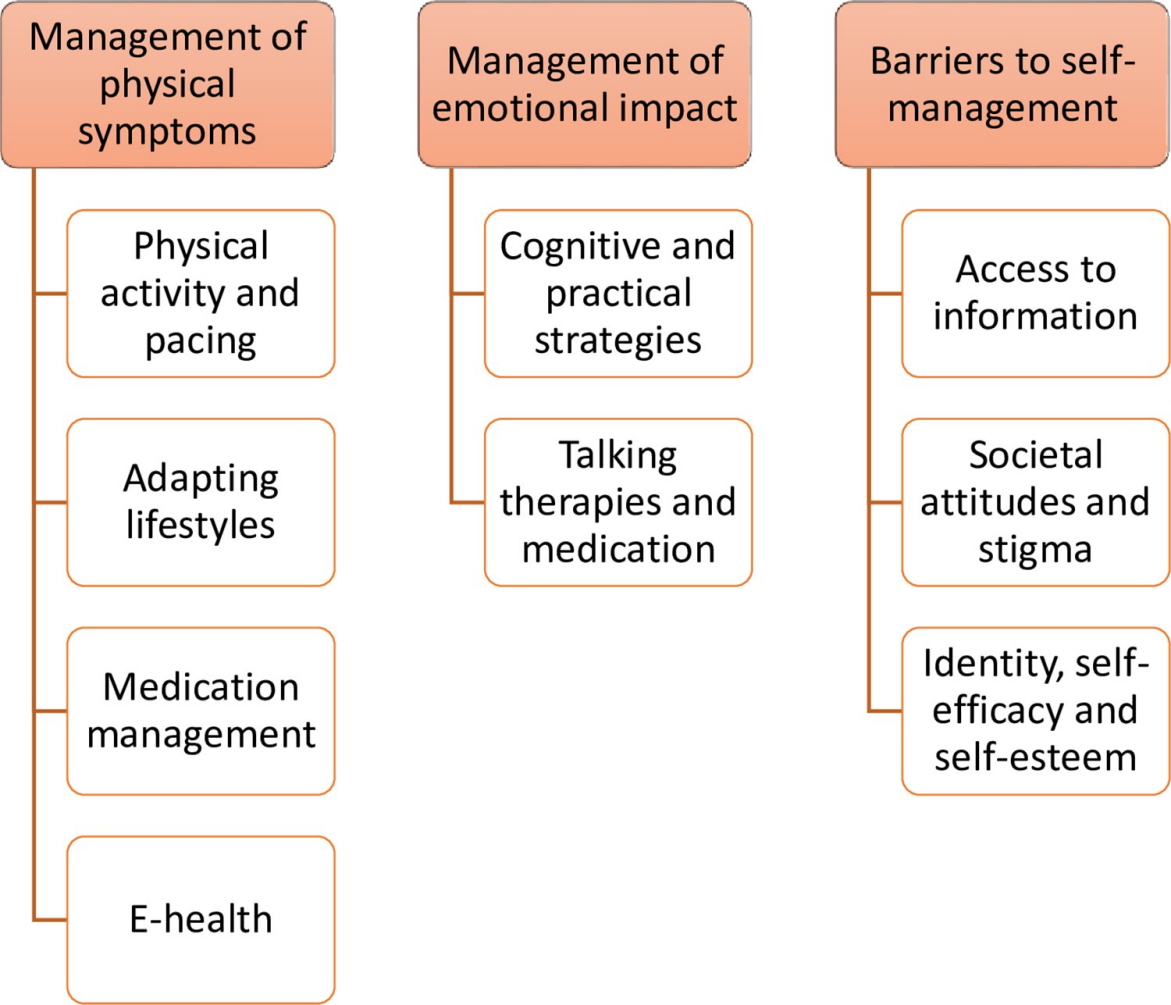
Themes, sub-themes, and codes from a thematic analysis exploring self-management in people with Parkinson’s.

We identified three overarching themes of management of physical symptoms, emotional impact and barriers to self-management, each with a series of sub-themes. Detail of the main themes and sub-themes can be seen below in Fig 1.

### Management of physical symptoms

The participants discussed the ways in which they self-manage the range of symptoms they experienced day to day through their own activities and by managing their medication. This included both physical activity and pacing, adapting with lifestyle and tailoring their medication.

#### Physical activity and pacing

Some participants expressed involvement in physical activity to keep symptoms at bay:

*“I do a little exercise programme every morning in the hope it keeps some of the limbs moving more freely”*. [Male, age 79, 6 years post diagnosis]

*“But we do exercise*. *We do exercise–when [wife]’s arthritis permits*, *we do a bit of walking*. *Not heavy stuff or anything*, *sort of 1 or 2 miles*, *but enough to keep a few muscles going*..*”* [Male, age 74, 3 years post diagnosis]

Others highlighted that completing exercises could be challenging due to symptoms related to the Parkinson’s and to other conditions:

*“It’s basically lift your legs up*, *lifting the arms up*, *and moving your body around*. *It’s exercises which I was given by the physiotherapist*. *They’re helpful*. *They keep me mobile although it’s really inconvenient to do it because it’s painful*. *Along with Parkinson’s I have neuropathic pain in the feet and that’s my worst thing because it makes me immobile*. *I can’t walk on my feet*. *I got to do it*, *otherwise I will become… It will all freeze up*.*”* [Male, age 82, 12 years post diagnosis]

However, many participants also discussed pacing their activities, including rest breaks or not partaking in many strenuous activities throughout the day:

*“Any tummy pains I have or any headaches I might have*, *they seem to abate*. *Lying down and resting works for me”*. [Male, age 75, <1 year diagnosis]

When participants were asked about what would be beneficial in a self-management resource, exercise tips and videos was frequently suggested:

*“Obviously*, *general fitness is very useful*, *and Pilates classes*, *I think*, *are good*. *Qigong*, *tai chi*, *those kinds of things are very useful for stimulating your body and*, *how can I put this*, *strengthening your mind*, *in a way*.*”* [Male, 67, 3 years post diagnosis]

#### Adapting lifestyles

Most participants discussed adapting their lifestyles and/or using environmental modifications to make living with Parkinson’s easier for them, although some found this frustrating and potentially costly:

*“So*, *we do all our shopping online now and have it delivered*. *It’s ridiculous because I’ve shopped for years*. *There’s no reason why I should be*. *So*, *that’s why I think it’s just the Parkinson’s that I get this anxiety*.*”* [Female, age 75, 6 years post diagnosis]

*“I’ve got an egg boiler now*. *I’ve got a tilting kettle to make myself a cup of tea with*. *Once again*, *all these things are expenses that I have to shell out for to make myself independent”*. [Male, age 71, unknown date of diagnosis]

One participant reached out to their local Parkinson’s society to enquire about the financial aspect of purchasing aids:

*“I’ve got the number of the lady who runs the local Parkinson’s Society*, *and I wanted to ask her a question about VAT*. *When we go to the mobility shop in [name of city] to buy things like walking sticks and wheelchairs*, *he doesn’t charge us VAT*.*”* [Male, age 82, 7 years post diagnosis]

One participant discussed seeking out complementary therapies to help with symptom management:

*“It helped massaging the feet*. *Obviously*, *it has a psychological effect*, *it’s been cured*, *for a day or two I didn’t feel anything*. *Less pain*, *I did feel that but less pain than normal*. *So it was worthwhile*. *Spending the money*, *spend on massage and so on*.*”* [Male, age 82, 12 years post diagnosis]

#### Medication management

Most participants accepted taking medication for their Parkinson’s was a new part of their life and daily routine. Many however found it difficult to adjust indicating more support with medication management including discussing the benefits is needed:

*“For somebody who has always resisted taking medication for anything throughout their life*, *the fact that I have to take medication three times a day*. *Is that challenging*? *It’s been quite revelatory for me”*. [Male, 67, 3 years post diagnosis]

With the complexity of medication management in Parkinson’s to ensure sustained effectiveness, remembering to take tablets throughout the day was crucial and some used aids for this:

*“They made sure that you had your tablets on time by setting alarms”*. [Female, age 74, 8 years post diagnosis]

The side effects associated with Parkinson’s medications were discussed, with mention of participant’s contacting their doctors to have their medication or dosage changed. However, one participant spoke of altering their dosage themselves without seeking medical advice:

*“Taking these tablets four times a day with all the others I’ve got to take*. *I can’t take it*. *It made me feel ill*. *So*, *I stopped it*. *I’m just taking them three times a day and that’s suits me”*. [Female, age 73, 7 years post diagnosis]

A few participants highlighted that the lack of information on medication was an issue and they would like to know more about the various options available to them:

*“I think one thing that I found not a lot of good at all is the one where they discuss the pros and cons of various drugs*. *I find it very difficult to get any sort of information about the drugs*. *And their quirks and things that they’re good at and the things they’re not good at*. *You don’t always get good information on that*.*” [*Male, age 74, 3 years post diagnosis]

Having access to accurate information about medication was flagged up as important to include in self-management resources:

*“I think it’s also important to have quite clear explanations of medication and what the implications are as well*.*”* [Male, age 67, 3 years post diagnosis]

#### E-health

Many participants were keen users of the internet to increase their knowledge on Parkinson’s, either regarding symptoms, ways to manage their condition, or medical advice. A few participants added that their family and caregivers researched ways to cope with Parkinson’s. While many participants would use websites such as Parkinson’s UK for information, some expressed concern using the internet.

The consistency of online advice has been said to vary which worried some participants:

*“I could type my medication into Google and come up with different health information on that particular medicine from different health authorities around the world*. *They’re all different*, *it’s all different”*. [Male, age 74, unknown date of diagnosis]

It was also found that some participants experienced physical barriers to accessing E-Health due to their condition; symptoms such as tremors inhibited the use of technological devices to access the internet:

*“I press the wrong buttons and Parkinson’s doesn’t help because my hands tremor is such that I can press the wrong button very easily and I do*. *So it’s quite difficult sometimes to do anything on the computer”*. [Female, age 85, 15 years post diagnosis]

A few participants expressed concern about having to access any self-management support online due to not having the technology:

*“We don’t have online so obviously keep in mind that not everybody has a computer*. *I don’t have a magic phone or any of those*.*”* [Male, age 82, 7 years post diagnosis]

Although forums may provide a degree on anonymity, some still felt uncomfortable using them for support as it required speaking opening about their condition:

*“I’m not very good at using forums*, *because you have to expose yourself to the rest of the world and I prefer to keep that sort of thing*, *under lock and key*. *I’m very controlled about what information I let out”*. [Male, age 74, 3 years post diagnosis]

### Management of emotional impact

Living with Parkinson’s had a wide-ranging emotional impact, which influenced individuals’ self-management of their condition and was addressed using a range of approaches.

#### Cognitive and practical strategies

To cope with the emotional burden of the condition, a few participants spoke of turning to activities which they found relaxing or used as a distraction, such as baking, listening to music, or watching television:

*“Watching a film sometimes helps because you’re into the film*. *And you can ignore the fact of how you’re probably feeling”*. [Male, age 75, <1 year post diagnosis]

A few participants also spoke of keeping themselves busy to keep active, and distract them from overthinking about their condition:

*“I don’t sit and mope about it*. *I say*, *come on*, *let’s go out somewhere*. *Just to keep exercising*. *So*, *it takes your mind off things”*. [Female, age 73, 7 years post diagnosis]

Some participants developed their own cognitive strategies to manage low mood or anxiety symptoms. There were some who actively thought of previous encounters which made them happy and lifted their mood:

*“I sit down and think about*, *a little bit*, *think of the happy days and happy…the trouble-free problems”*. [Male, age 77, 6 years post diagnosis]

Others used techniques to manage their anxiety:

*“What I usually do is I’ll sit back and I’ll sort of [inhaling and exhaling] take deep breaths and then I just sort of think*, *‘calm down*, *just calm down’*, *you know*, *and sort of bring myself back again*.*”*. [Female, age 75, 6 years post diagnosis]

#### Talking therapies and medication

A few participants were prescribed medication to manage their emotional symptoms indicating the range of techniques participants use.

*“I take my antidepressants in the morning*. *And when I’m by myself I’ll have a little cry*. *So that’s how I manage my moods”*. [Male, age 71, unknown date of diagnosis]

Access to talking therapies was highlighted as a need to help manage emotional burden. In particular a wish to be counselled specifically for Parkinson’s or by someone who has experience of Parkinson’s.

*“Counselling*, *somebody you could have–you do get general counselling*, *but I’d like counselling for Parkinson’s itself because then you could just go to that person and tell them all your problems*, *how you feel*, *how it’s hurting*, *how you don’t want to accept that you’ve got Parkinson’s*, *how your immediate family react to you*. *And you could just let your emotions out*. *And then they could understand as well what you’re going through*. *And they could maybe give you tips to manage it*.*” [*Male, age 71, unknown date of diagnosis]

One participant described taking up therapy to help manage the emotional burden of Parkinson’s, though this was not common:

*“Well*, *I did a very good CBT course and mindfulness*, *and I use a variety of techniques to unfreeze myself*.*”* [Male, age 62, 18 years post diagnosis]

### Barriers to self-management

Self-management happens within an individual’s contextual environment, which is influenced by societal attitudes, culture, health and social care systems and policy. In this study there were three main aspects that seemed to impact people’s lives and ability to self-manage: access to information, stigma, and the impact of this on their self-esteem and identity.

#### Access to information

Many participants expressed a lack of information or advice given to them by health care professionals.

*“Not really*. *Nothing really as powerful as advice… I mean the sort of the quality of the input from the doctor wasn’t at all–some of them are extremely poor communicators*.*”*. [Male, age 75, <1 year post diagnosis]

This lack of information and advice was particularly evident when diagnosed:

*“When I was first diagnosed*, *nobody really got in touch with me*. *I was just told I had Parkinson’s and that was it”*. [Female, age 75, 6 years post diagnosis]

*“No*, *no advice [given]*. *That’s why I didn’t get on with the person who diagnosed me at all*. *I mean*, *they don’t give you enough information*.*”* [Male, age 62, 18 years post diagnosis]

However, some felt overwhelmed with the information that was out there when they actively sort it:

*“[it is] too daunting*, *it was too sort of like*, *it was too scary to look at*, *thinking*, *you know it came up as you know brain surgery”*. [Female, age 47, 8 years post diagnosis]

One participant highlighted they would like any self-management resource to include signposting to organisations or people who can provide further information on Parkinson’s as well as being a resource for Parkinson’s itself:

*“Maybe some sort of phone numbers or whatever relevant departments where we can contact someone*, *in case of anybody with this sort of condition*. *Maybe there can be some handy stretching*, *some leaflets or some knowledge on stretching exercises which could be helpful as well*.*”* [Male, age 45, 8 years post diagnosis]

#### Societal attitudes and stigma

For some participants it was clear that their perception of negative attitudes towards Parkinson’s or misinterpretation of the symptoms of Parkinson’s influenced their confidence and ability to self-manage.

A few participants raised that Parkinson’s is still not well understood by the general public, which leads to increased stigma:

*“Parkinson’s is overlooked*, *and I think somehow you have to build confidence in people because out and about people stare at you if you’re shaking”*. [Female, age 75, 15 years post diagnosis]

The consequences of stigma, led to many participants avoiding public spaces due to feeling embarrassed:

*“I tend to stick to my room*, *rather than go anywhere*. *I feel a little bit embarrassed as well to go out because I feel low”*. [Male, age 45, 8 years post diagnosis]

Moreover, as Parkinson’s usually affects people over the age of 50, those diagnosed with the disease at a younger age felt noticed more in society and experienced greater stigma:

*“When I got to my local hospital*, *it’s terrible*. *They’re sort of like–you see them whispering*, *“she’s got Parkinson’s”*. *And because I’m young*, *that’s a stigma for me Asking for that [help]*, *sometimes it’s hard thing to ask for that*, *“Can you help me do this*?*” because they look at you and think you look absolutely fine*.*”*. [Female, age 47, 8 years post diagnosis]

#### Identity, self-efficacy, and self-esteem

Many participants’ ability to self-manage their condition appeared to be influenced by their outlook on life and whether they accepted having Parkinson’s as part of their identity or not. Those who appeared more accepting of Parkinson’s and where it had not impacted their self-esteem or self-efficacy typically seemed to manage better.

*“I know that it’s effectively a degenerative condition and things are not going to get better*, *they’re going to get worse*, *but there’s no point in worrying about it”*. [Male, age 67, 3 years post diagnosis]

Some participants had clearly accepted they had Parkinson’s and did not fight the ‘Parkinson’s identity’, which seemed linked to their outlook being more positive overall.

*“The way I deal with it with in circles where I know people*, *like the neighbours and church and friends and acquaintances*, *is I’m very open about it and tell everybody exactly what’s going on*. *I find that much easiest*. *Because people then look through it… I mean*, *your close friends and family just look through it*, *just see me still*. *I decided to be open straightaway*.*”* [Male, age 62, 18 years post diagnosis]

However other participants indicated a wish to accept the Parkinson’s, but later in the interview indicated that they were still negatively affected emotionally by the impact of having Parkinson’s:

*“You have to kind of make friends with the Parkinson’s rather than let it be an enemy…You go negative*, *because your life’s changed and you’ve become distraught*, *you become negative*. *Nothing*, *there’s not positives left”*. [Female, age 47, 8 years post diagnosis]

*“I mean it’s really depressing to think that you’re not the same person that you used to be*. *And I know people say*, *“You’ve got to rise above it*, *you are*, *you’re still [name] and you have to*…*”*, *but you’re not*. *The truth hurts*, *but it’s the truth*, *you’re not the same person*. *You do change*, *socially*, *mentally*. *And a lot of them tell you that time and time again as well*.*”* [Female, age 47, 8 years post diagnosis]

The ability to effectively self-manage is linked to people’s self-esteem and self-efficacy. Many expressed that Parkinson’s had impacted their self-esteem and self-efficacy:

*“I tend to stick to my room*, *rather than go anywhere*. *I feel a little bit embarrassed as well to go out*, *because I feel low*. *My… What I would say*? *My self-esteem*, *I’m more concerned about what others would think about me when I’m walking as well*. *But slowly*, *slowly I’m getting used to it*. *My workplace doesn’t know that I’m having this condition*. *Until now*.*”* [Male, age 45, 8 years post diagnosis]

*“Well I’m constantly scared of falling*, *doing things like the washing*, *you’ve got to bend over to get into the washing machine*, *bend over to get into the tumble dryer*.*”* [Male, age 71, date of diagnosis unknown]

*“So*, *we went to the charity shop with that and we went to the bank…Then my husband said*, *what should we do*? *I said*, *“no*, *I’ve got to go back now”*. *I’d had enough*. *My legs get very shaky and I get more nervous*. *I’m okay one minute and then it’ll start building up*. *And then that was it*. *We did those three things and we had to go back to the car and come home…I think it’s just anxiety*. *It’s not that I’m frightened of anything or nervous about anything*, *but the anxiety*.*”* [Female, age 75, 6 years post diagnosis]

Some felt frustrated at being helped too much and had a sense of losing their independence and self-efficacy.

*“‘Don’t do this*, *you’ll fall*!*’ I do get sometimes impatient with being helped so much*. *There are times when I need to be independent*. *There are also times when I need the wheelchair to be brought up*, *and help given*. *But I get annoyed if I feel that I’m not being given the chance to do things independently*. *Because I don’t mind if something happens*, *so long as I die as a result in a not too painful way*. *And I’d rather that*, *than be mollycoddled*, *I think*, *instead*.*”* [Female, age 85, 15 years post diagnosis]

One participant of Asian ethnicity spoke of the cultural views associated with Parkinson’s, showing how culture ideologies and norms play a role in stigmatizing certain illnesses:

*“And there is a stigma attached to the condition back home*, *where I come from*. *Wherein*, *no one tends to divulge–well actually it’s a private information*, *like my condition*. *So nobody would like*, *even my family wouldn’t like to tell anybody else that I’m having this condition*. *So it’s better off taking me as a drunk*, *rather than a PD patient*.*”* [Male, age 45, 8 years post diagnosis]

## Discussion

This study identified the ways in which people with Parkinson’s self-manage their condition, and what helped and hindered this. Participants used a range of strategies to support them in ‘living well’ with Parkinson’s including pacing and physical activity, lifestyle adaptations and aids, medication management, focusing on enjoyable activities and hobbies or those that distracted them, cognitive strategies for low mood and anxiety and accessing professional help e.g., talking therapies. Access to information and advice is a large part of self-management and participants felt left on their own to access this with little support from health care professionals. This was particularly evident at around the time of diagnosis. All the self-management was set within the context in which the participants were living, and this included for some feeling stigmatised by the public, which limited their activities.

Management of complex medication regimes is a key part of self-management of Parkinson’s. For this, they need access to accurate information, timely specialist advice as well as practical support such as devices that remind participants to take their medication. Alongside medication there are also practical activities people with Parkinson’s can do to elevate physical symptoms such as use of exercise [20]. However, not all participants discussed using exercise to manage or help ease symptoms. Overall, when participants were asked to discuss ways in which they self-managed many found it difficult to thinks of ways in which they did this.

Parkinson’s can have a large impact on the emotions and mental health of those affected and finding ways to cope with this are important [21]. Some participants felt stigmatised by the public, which lead to less engagement in beneficial activities such as going for a walk. Studies have found people with Parkinson’s to have been mislabelled as alcohol dependent [22]. Participants expressed the need to avoid public spaces due to stress, which has been found to worsen symptoms of Parkinson’s and reduce the effect of medications [23]. People’s self-esteem and self-efficacy were impacted leading to avoidance of certain activities such as leaving the house. This appeared to be influenced by how participants identified with their Parkinson’s; those who accepted their Parkinson’s appeared to have less psychological distress. Research has shown within multiple sclerosis (MS) that identifying as ‘someone with MS’ predicted lower anxiety and depression and higher help seeking behaviours [24]. Support with managing and coming to terms with Parkinson’s is likely to be beneficial for the person’s wellbeing.

Accessing information on what might be best for emotion and physical symptoms was challenging for participants. Some used online to help them find accurate information, but this led to some feeling overwhelmed. Research has shown that the older population who need support self-managing symptoms may be best done where they can access the information with ease and are unlikely to feel stigmatised [25]. Our findings support that people may not actively self-manage if they feel they may be stigmatised and therefore support accessing information in the home (e.g., online) may be beneficial. Furthermore, consistent with previous research [26], our findings also suggested participants would like to access information through their health care professionals as opposed to seeking it themselves. However, many felt health care professionals did not provide information, either it was not offered or they felt the health care professional lacked understanding of the condition, which was particularly evident when first diagnosed with Parkinson’s.

### Future research and implications for clinical practice

Our study has highlighted several important self-management components for people with Parkinson’s living in the UK. These components include access to accurate information about Parkinson’s such as medication and emotional support, individualised planning of exercise regimes, and support when newly diagnosed particularly around managing the Parkinson’s stigma. Holistic and person-centred self-management programmes or interventions should be developed to test these components for effectiveness and adoption by the NHS. Furthermore, providing more public health knowledge on Parkinson’s will allow people to be more aware of the condition thus aiming to reduce stigma.

### Strengths and limitations

We identified a range of self-management strategies from a diverse group of people living with Parkinson’s across the disease spectrum from newly diagnosed to >20 years since diagnosis. This allowed understanding of the varying ways which disease progression of Parkinson’s may affect self-management. Our sample had fewer people from ethnic minority groups, and those living in deprived areas, and were recruited from in and around the London region, which may not reflect the experiences and attitudes of those in other areas and across underrepresented groups.

## Conclusion

This study has found people with Parkinson’s within the UK currently self-manage in a range of ways such as incorporating physical activity, cognitive strategies, talking therapy and medication into their lives. However, each of these has their own barriers such as lack of access to information or struggling to use e-health, challenges remembering to take medication or engage in activities, and the financial impact of certain aids and therapies. In addition to this some people with Parkinson’s felt stigmatised by the public, which impacted their self-esteem and willingness to embrace being considered ‘someone with Parkinson’s’. This study has identified a range of areas that should be incorporated into future self-management interventions or resources to support people with Parkinson’s maintain their independence.
